# Pediatric peritoneal dialysis in Brazil: a discussion about sustainability. A document by the Brazilian Society of Nephrology, the Brazilian Society of Pediatrics, the Brazilian Association of Organ Transplantation, and the Brazilian Association of Dialysis and Transplant Centers

**DOI:** 10.1590/2175-8239-JBN-2021-0245

**Published:** 2022-03-28

**Authors:** Lilian Monteiro Pereira Palma, Maria Goretti Moreira Guimarães Penido, Nilzete Liberato Bresolin, Marcelo de Sousa Tavares, Lucimary Sylvestre, Olberes Vitor Braga de Andrade, Rejane de Paula Bernardes, Maria de Fátima Santos Bandeira, Clotilde Druck Garcia, Vera Hermina Kalika Koch, Vera Maria Santoro Belangero, Anelise Uhlmann, Emília Maria Dantas Soeiro, Arnauld Kaufman, Maria Cristina de Andrade, Roxana de Almeida Roque Fontes Silva, Viviani Calice-Silva, Marcos Alexandre Vieira, Osvaldo Vieira Merege

**Affiliations:** 1 Universidade Estadual de Campinas, Departamento de Pediatria, Campinas, SP, Brasil.; 2 Unidade de Nefrologia Pediátrica do Centro de Nefrologia da Santa Casa de Belo Horizonte, Belo Horizonte, MG, Brasil.; 3 Universidade Federal de Santa Catarina, Florianópolis, SC, Brasil.; 4 Hospital Pequeno Príncipe, Curitiba, PR, Brasil.; 5 Faculdade de Ciências Médicas da Santa Casa de São Paulo, São Paulo, SP, Brasil.; 6 Clínica Nefrokids Curitiba, Curitiba, PR, Brasil.; 7 Unidade de Nefrologia Pediátrica NefroClínicas, Rio de Janeiro, RJ, Brasil.; 8 Universidade Federal de Ciências da Saúde de Porto Alegre, Serviço de Nefrologia Pediátrica da Santa Casa de Porto Alegre, Porto Alegre, RS, Brasil.; 9 Instituto da Criança e Adolescente do HCFMUSP, São Paulo, SP, Brasil.; 10 Hospital Criança Conceição, Porto Alegre, RS, Brasil.; 11 Instituto de Medicina Integral Professor Fernando Figueira, Recife, PE, Brasil.; 12 Universidade Federal do Rio de Janeiro, Instituto de Puericultura e Pediatria Martagão Gesteira, Hospital Federal dos Servidores do Estado, Rio de Janeiro, RJ, Brasil.; 13 Universidade Federal de São Paulo, Escola Paulista de Medicina, São Paulo, SP, Brasil.; 14 Assistência Médica Infantil da Paraíba, João Pessoa, PB, Brasil.; 15 Universidade da Região de Joinville, Fundação Pró-Rim, Joinville, SC, Brasil.; 16 Universidade de São Paulo, Faculdade de Medicina de Ribeirão Preto, São Paulo, SP, Brasil.

**Keywords:** Peritoneal Dialysis, Child, Adolescent, Single-Payer System, Sustainable Development Indicators, Diálise Peritoneal, Criança, Adolescente, Sistema de Fonte Pagadora Única, Indicadores de Desenvolvimento Sustentável

## Abstract

**Introdução:**

A diálise peritoneal (DP) é importante para a pediatria. Este estudo mostrou dados de centros brasileiros que utilizam DP pediátrica.

**Método:**

Estudo transversal, observacional, descritivo com questionário eletrônico. Incluiu-se pacientes de 0-18 anos em DP cadastrados nos bancos de dados dos diversos centros. Questionário preenchido anonimamente, sem dados de identificação. Foi adotada metodologia quantitativa.

**Resultados:**

212 pacientes estão em DP no Brasil (agosto, 2021). 80% têm menos de 12 anos de idade. A maioria realiza DP automatizada e 74% são dependentes do Sistema Único de Saúde. Em 25% dos centros faltou material de DP e em 51% os pacientes pediátricos foram convertidos de DP para HD.

**Conclusão:**

A maioria dos pacientes tinha menos de 12 anos e era dependente do SUS. A escassez de insumos aconteceu em 25% dos centros. Esses dados apontam para o problema da sustentabilidade de DP, única alternativa de TRS em crianças muito pequenas.

## Introduction

Acute kidney injury (AKI) and chronic kidney disease (CKD) are conditions commonly seen at pediatric tertiary referral centers. Treatment includes conservative measures and renal replacement therapy (RRT)^1^. Patients with CKD are treated by physicians from different medical specialties including cardiology, endocrinology, neurology, pulmonology, and cardiac and vascular surgery, to name a few. Patients with stage 5 CKD are required to undergo RRT and are at risk of dying^1^. According to the latest census survey of the Brazilian Society of Nephrology, the estimated global prevalence of patients on chronic dialysis moved from 405 pmp in 2009 to 640 pmp in 2018, an absolute increase of 58%, which corresponds to an average increase of 6.4% per annum. Most prevalent patients (92.3%) were on hemodialysis (HD) and 7.7% were on peritoneal dialysis (PD).

The treatment of acute and chronic kidney dysfunctions has been the subject of intense discussion, particularly in matters concerning RRT, which includes PD, intermittent and extended HD, and continuous blood purification methods^2-4^. Recent studies shed light on the shortage of dialysis supplies in underdeveloped and developing nations^5^. Brazil presents a low overall prevalence of children on chronic dialysis, as seen in other nations with a similar socioeconomic profile, along with substantial discrepancies between regions in the country when chronic dialysis for pediatric populations is considered^6^. According to the Brazilian Census Bureau (IBGE), 71.5% of the Brazilian population relied on the Brazilian Public Healthcare System (SUS) for medical treatment in 2019.

A less-than-ideal supply of PD to pediatric patients is a death warrant for many children who have not achieved the minimum body weight required to undergo HD. Newborns and infants aged a few months or with low weight, extreme preterm babies, and neonates with congenital kidney or urinary tract conditions with CKD have in PD their only chance to survive until a kidney transplant is performed.^7^ In addition, PD is advantageous to pediatric patients when compared to HD for a number of reasons, which include lower chance of suffering growth failure and losing residual kidney function, and preservation of vascular access points^7^. Financial impact is also proportionately smaller. And last but not least, patients who live far from care centers and individuals unable to find a time slot in HD shifts are also directed to PD^6^.

Considering the positive impact of PD, this study aimed to identify the number of RRT centers in Brazil that offer PD to pediatric populations, the number of patients on PD, the main payment source, and the number of patients on PD who had to switch to HD.

## Patients and Methods

### Study Design

This is a cross-sectional observational descriptive study.

### Patients

The study included pediatric patients on PD diagnosed with CKD aged 0-18 years registered with a number of RRT centers in Brazil. Patients aged 18 and older were not included.

## Methods

A questionnaire posted on platform SurveyMonkey containing ten questions about PD in Brazil (https://pt.surveymonkey.com/r/CPBMCJ3) ^8^ was broadly disseminated via electronic media, personal calls, and social media platforms. The questionnaires were answered voluntarily and did not capture personal respondent data (Annex 1). A quantitative methodology was adopted and the following variables were captured: number of participating centers; number of patients; PD type; need to switch from PD to HD; payment source for PD supplies; and whether supply shortages occurred within the last six months.

### Statistical Analysis

Statistical analysis was performed on platform SurveyMonkey. Quantitative variables were expressed as absolute and percent frequencies.

### Ethics

The study was conducted in accordance with the standards stipulated in the Declaration of Helsinki of 1964. Informed consent terms were not required, since patients were not asked to send in personal identification information and remained anonymous. Only numerical data from the care center databases were used.

## Results

**Figure 1:**
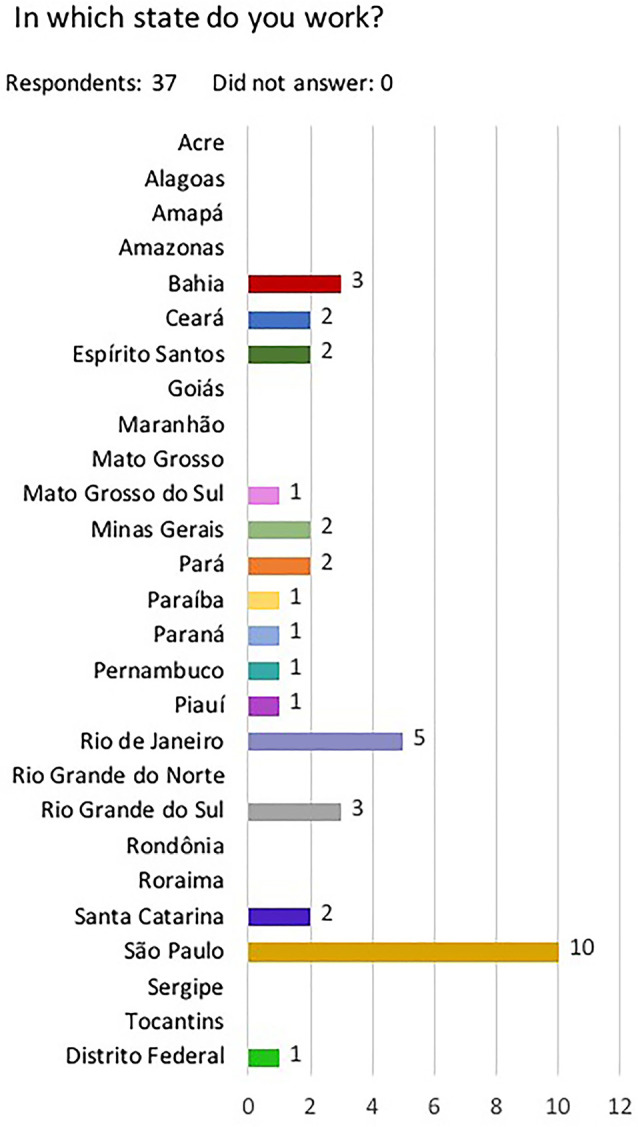
Number of dialysis centers with patients aged 0-18 years on peritoneal dialysis per State in Brazil.

**Figura 2:**
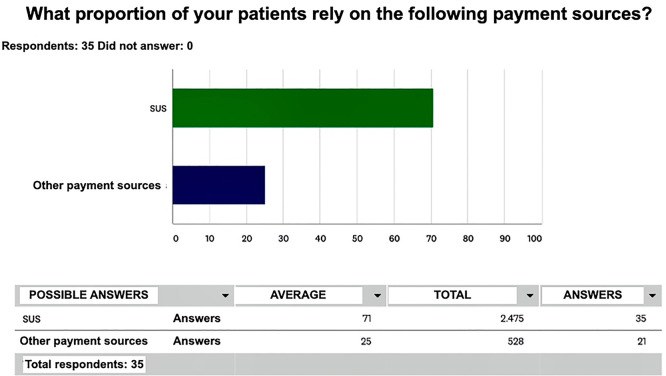
Em 74% dos casos, a fonte pagadora das máquinas e insumos de diálise peritoneal pediátrica no Brasil é o Sistema Único de Saúde (SUS).

A total of 60 care centers (Figure 1) answered the questionnaire. In 23 centers PD was either not performed at all or was not prescribed to patients aged less than 18 years. The data from 37 centers (Table 1) and their 212 pediatric patients on PD at the time of the study were analyzed. A total of 175 patients (83%) were aged 0-12 years and the remaining 37 (17%) were aged >12 and <18 years. Automated PD (APD) was the most common treatment mode (86%) and the SUS stood as the most common payment source. Baxter^®^ was the main equipment and supply provider for pediatric PD in Brazil, present in more than 80% of the care centers (Figure 2). A quarter (25%) of the care centers reported supply shortages within the last six months. Need to switch patients from PD to HD was a reality in 19 (51%) of 37 care centers.

**Table 1:** The 37 centers with pediatric pd programs in Brazil that answered the questionnaire

State	Center	State	Center
SP	Santa Casa de São Paulo	SP	Hospital de Clínicas - FMRP - Ribeirão Preto
SP	Hospital de Clínicas Unicamp	BA	Hospital Ana Nery
MG	Santa Casa de Belo Horizonte	DF	Hospital da Criança de Brasília
RJ	Hospital Federal de Bonsucesso	PR	Hospital Pequeno Príncipe/Raul Carneiro
PA	Hospital Regional do Araguaia	SP	DaVita São José dos Campos
PE	IMIP - Instituto de Medicina Integral Professor Fernando Figueira	MS	HUMAP - Hospital Universitário Maria Aparecida Pedrossian UFMS/EBSERH
ES	HUCAM/UFES/EBSERH - Hospital Universitário Cassiano Antônio Moraes	ES	Instituto Capixaba do Rim
PA	Santa Casa do Pará	PB	AMIP - Assistência Médica Infantil da Paraíba
SP	Instituto da Criança HCFMUSP	MG	
SP	UNIFESP	SP	Hospital de Base São José do Rio Preto
CE	DaVita	SP	Hospital Infantil Sabará
RS	Hospital de Caridade de Ijuí	RJ	Hospital Infantil Pedro Ernesto
RJ	Prontobaby	RS	Santa Casa de Porto Alegre
	IPPMG - Instituto de Puericultura e Pediatria Martagão Gesteira, UFRJ		
	Hospital Jesus		
RJ	Nefroclínicas Ipanema	CE	DaVita Meireles
BA	Clínica Senhor do Bonfim	SP	HIAE - Hospital Israelita Albert Einstein/UNIFESP/Vila Nova Star
PI	Clinefro	SC	Centro de Tratamento de Doenças Renais de Joinville
SC	Hospital Joana de Gusmão	BA	Fresenius Ined
RS	Hospital Geral Caxias do Sul	SP	UNESP Botucatu

## Discussion

This study shed light on the reality of pediatric PD in Brazil. Data from the Brazilian Transplant Registry show that 305 patients aged less than 18 years were on the waiting list for a kidney transplant in June 2021. Our study found that most of them were on PD.

PD remains the main therapy to treat acute and chronic kidney dysfunction, particularly for small children living in underdeveloped and developing nations. Raina et al. indicated that the scarcity of financial and medical resources, trained interdisciplinary teams, and support funds have prevented the establishment and maintenance of proper care standards in countries facing unfavorable economic conditions^8^. In a study about AKI, Smoyer et al. found that in nations facing challenging socioeconomic conditions, acute kidney injury is a frequently fatal disease associated with myriad conditions such as malaria, leptospirosis, volume depletion (acute gastrointestinal symptoms, obstetric hemorrhage), and nephrotoxicity^9^. The authors also stressed that cases of AKI, particularly in patients with oliguria and hydroelectrolytic and acid-base disorders, may be mostly treated with PD^9^. In a recent guideline document, Nourse et al.^10^ stated that PD might be the best option for small newborns with cardiovascular instability, particularly in places where continuous blood purification methods are not available.

PD is a low cost mode of dialysis known for it simplicity and application in hemodynamically unstable patients. Nevertheless, PD is not available in many parts of the world. A large population study showed that pediatric patients with AKI suffer with higher death rates in countries with medium-to-low levels of per capita income^11^. On the other hand, although HD is a rather efficient method in managing the metabolism of patients with acute kidney injury, it requires trained teams and often expensive equipment, a tall order in places with few resources and poor access to treated water and electricity. Besides, HD might not be suitable for hemodynamically unstable patients. The best option for such patients is continuous RRT (expensive, requires treated water and electricity, and expertise from the healthcare workers assisting in the process and nursing staff)^12^.

Brazil has recently seen a decrease in the availability of PD. Causes include low fees paid to suppliers, insufficient compensation paid to healthcare workers, lack of trained personnel, and shortage of spare supplies. These circumstances have led to the prescription of HD to children who otherwise would have been on PD. Many families travel for hundreds of miles to the nearest care center so that their children are given proper treatment^13^. Parents have to miss work to care for their children and often lose the very jobs they need to provide for their families. Family dynamics changes, children miss school, and issues with vascular accesses compound to build hurdles to patient progression and draw them closer to a kidney transplant^14^. Death might be the outcome in places where PD or HD are not available and for newborns and children who live in cities where HD is the only option^15^.

## Conclusions

A significant number of pediatric patients are currently on PD in Brazil. More than 80% are aged less than 12 years and most rely on APD. The main payment source for treatment is the SUS (76%) and the main provider of equipment and supplies is Baxter^®^. Fresenius^®^ is no longer accepting patients from the SUS on account of the low prices paid for equipment and supplies.

Some centers have more than 90% of their pediatric patients on HD due to difficulties with supporting SUS-funded PD programs. In 19 centers (51%), patients had to switch from PD to HD. This may clearly result in not offering treatment to small children on account of difficulties related to vascular access, poor access to trained healthcare workers, and lack of proper equipment. Absence of treatment for patients in need of RRT is a death warrant.

Currently, there is a significant number of pediatric patients on PD in Brazil. More than 80% are children under 12 years old, the majority being APD dependent. The main source of payment is the SUS (76%), whose input supplier is mostly Baxter^®^. This result is also due to the fact that Fresenius^®^ ended the inclusion of new patients by the SUS due to the price gap.

There are centers with more than 90% of children on HD due to the difficulty of maintaining the PD program by the SUS. In 19 centers (51%) there was a need to convert patients from PD to HD. Clearly, this can result in the inability to maintain treatment in young children due to difficulty in vascular access, untrained professionals, and inadequate equipment. Therefore, the absence of treatment for patients who need RRT represents a death sentence.

The Brazilian Society of Nephrology (SBN) has 3,500 members and the Brazilian Society of Pediatrics (SBP) has more than 25,000 members. In both Societies, courses on pediatric nephrology are frequently designed for pediatricians who care for children in different regions of Brazil. Some courses include hands-on activities (Hands on Courses).

The “Saving Young Lives” initiative, a partnership between the International Society of Pediatric Nephrology and the Latin American Association of Pediatric Nephrology, makes it possible to include (free of charge) two pediatric nephrologists from Brazilian regions with few resources for intensive training (theoretical and practical) in PD.

In addition to continuing education, it is very important that training services in Nephrology and Pediatric Nephrology have the modality in the service or that they enable training in some place that has it. PD training is part of the residency matrix and, currently, in Brazil there are 723 Hemodialysis units and 688 services that provide PD.

In December 2021, after many years of attempts, there was a 24% readjustment for PD supplies (without readjusting medical fees). In this way, there is still a need for great effort so that the doctor is remunerated in a dignified way.

Finally, there is a great deal of awareness-raising work to be carried out in consonance with the health authorities, seeking the involvement of the World Health Organization, as well as government officials, societies of medical specialties and multidisciplinary teams^6^.

Lastly, significant awareness building with the aid of health authorities and the involvement of the World Health Organization, Government, medical associations, and multidisciplinary teams is needed^6^.

## Supplementary Material

The following materials pertaining to this paper are available online:

Annex
