# Supplementary material for: Pediatric peritoneal dialysis in Brazil: a discussion about sustainability. A document by the Brazilian Society of Nephrology, the Brazilian Society of Pediatrics, the Brazilian Association of Organ Transplantation, and the Brazilian Association of Dialysis and Transplant Centers
Source: J Bras Nefrol. 2022 Mar 28;44(4):579–84. doi: 10.1590/2175-8239-JBN-2021-0245 (PMC9838676; doi:10.1590/2175-8239-JBN-2021-0245)
Supplement: Annex [file 2175-8239-jbn-2021-0245-s1.pdf]

Brazilian Journal of Nephrology
https://doi.org/10.1590/2175-8239-JBN-2021-0245

Supplementary Material to paper "Pediatric peritoneal dialysis in Brazil: a discussion about sustainability. A document by the Brazilian Society of Nephrology, the Brazilian Society of Pediatrics, the Brazilian Association of Organ Transplantation, and the Brazilian Association of Dialysis and Transplant Centers"

Annex

INITIAL SURVEY ABOUT PEDIATRIC PERITONEAL DIALYSIS IN BRAZIL – CHRONIC PROGRAM (developed on platform SurveyMonkey)

Pediatric PD in Brazil

This questionnaire was developed as part of an initiative held by the Brazilian Society of Nephrology and the Brazilian Society of Pediatrics. It was designed to map out care centers in Brazil providing CHRONIC peritoneal dialysis services to children and adolescents. The data provided may be used in documents designed to discuss the sustainability of Renal Replacement Therapy with Health authorities. PLEASE FILL ONLY ONE QUESTIONNAIRE PER CENTER. Thank you!

1. In which Brazilian State is your center located?

Acre

Alagoas

Amapá

Amazonas

Bahia

Ceará

Espírito Santo

Goiás

Maranhão

Mato Grosso

Mato Grosso do Sul

Minas Gerais

Pará

Paraíba

Paraná

Pernambuco

Piauí

Rio de Janeiro

Rio Grande do Norte

Rio Grande do Sul

Rondônia

Roraima

Santa Catarina

São Paulo

Sergipe

Tocantins

Distrito Federal

2. What is the name of your dialysis center?

3. Does the center where you work provide care to patients aged < 18 years in need of chronic peritoneal dialysis?

Yes No

4. If your answer to Question 3 was Yes, how many patients aged 0-12 years are on chronic hemodialysis in your center?

5. If your answer to Question 3 was Yes, how many patients weighing < 15 kg are on chronic peritoneal dialysis in your center?

6. What is the main mode of chronic peritoneal dialysis your center offers to pediatric patients?

Continuous ambulatory peritoneal dialysis (CAPD)

Automated peritoneal dialysis (APD)

7. What proportion of your patients rely on the following payment sources?

SUS

Other payment sources

8. In regard to access to chronic peritoneal dialysis to children, indicate the proportion of patients prescribed the following therapies:

CAPD

APD

9. How many patients aged less than 18 years on chronic peritoneal dialysis at your center reside in another municipality?

10. How many pediatric patients seen at your center have applied for a kidney transplant?
